# Alzheimer’s genetic risk effects on cerebral blood flow across the lifespan are proximal to gene expression

**DOI:** 10.1016/j.neurobiolaging.2022.08.001

**Published:** 2022-08-07

**Authors:** Hannah Chandler, Richard Wise, David Linden, Julie Williams, Kevin Murphy, Thomas Matthew Lancaster, Alzheimer’s Disease Neuroimaging Initiative (ADNI)

**Affiliations:** a Cardiff University Brain Research Imaging Centre (CUBRIC), School of Psychology, Cardiff University, Cardiff, UK; b Institute for Advanced Biomedical Technologies, Department of Neuroscience, Imaging and Clinical Sciences, G. D’Annunzio University of Chieti-Pescara, Chieti, Italy; c Department of Psychiatry and Neuropsychology, School for Mental Health and Neuroscience, Faculty of Health, Medicine and Life Sciences, Maastricht University, Maastricht, The Netherlands; d UK Dementia Research Institute, School of Medicine, Cardiff University, UK; e Cardiff University Brain Research Imaging Centre (CUBRIC), School of Physics and Astronomy, Cardiff University, Cardiff, UK; f Department of Psychology, University of Bath, Bath, UK

**Keywords:** Cerebral blood flow, Polygenic, Alzheimer’s disease, Gene expression, Lifespan

## Abstract

Cerebrovascular dysregulation such as altered cerebral blood flow (CBF) can be observed in Alzheimer’s disease (AD) and may precede symptom onset. Genome wide association studies show that AD has a polygenic aetiology, providing a tool for studying AD susceptibility across the lifespan. Here, we ascertain whether the AD genetic risk effects on CBF previously observed (Chandler et al., 2019) are also present in later life. Consistent with our prior observations, AD genetic risk score (AD-GRS) was associated with reduced CBF in the ADNI sample. The regional association between AD-GRS and CBF were also spatially similar. Furthermore, CBF was related to the regional mRNA transcript expression of AD risk genes proximal to AD-GRS risk loci. These observations suggest that AD risk alleles may reduce neurovascular process such as CBF, potentially via mechanisms such as regional expression of proximal AD risk genes as an antecedent AD pathophysiology. Our observations help establish processes that underpin AD genetic risk-related reductions in CBF as a therapeutic target prior to the onset of neurodegeneration.

## 1 Introduction

Variability of cerebrovascular function is heritable and partly explained by additive effects of genetic factors that converge across several neurobiological processes (Ikram et al., 2018). In Alzheimer’s disease (AD), cerebrovascular dysregulation is a key concomitant factor (Kelleher and Soiza, 2013), and is one of the earliest markers of AD pathophysiology (Iturria-Medina et al., 2016; Kelleher and Soiza, 2013). Decreases in cerebrovascular function are observed both in patients with AD and young individuals with an increased risk of dementia (Chandler et al., 2019; Filippini et al., 2011; Montagne et al., 2020; Wolters et al., 2017) This broadly suggests that altered cerebrovascular function is a risk factor for AD, rather than a consequence of the disease, which may be present across an individual’s lifespan.

Genome-wide association studies (GWAS) demonstrate that AD is also highly polygenic, where potentially thousands of common risk alleles confer susceptibility for disease (Kunkle et al., 2019). Although polygenic analysis has shown utility in predicting AD (Escott-Price et al., 2017; Escott-Price et al., 2015), the neurobiological mechanisms by which these loci confer risk remains poorly understood, particularly in relation to cerebrovascular function. Furthermore, the impact of these risk alleles across the lifespan has been seldom explored. Several studies have suggested that the influence of AD risk alleles may be age-dependent (Matura et al., 2016), while other large studies demonstrate that the impact of AD risk alleles on risk factors such as cognition are influential across the entire lifespan (Hill et al., 2016). However, the impact of AD risk alleles on *in-vivo* measures of brain function has not been investigated across the lifespan.

In our previous work we used arterial spin labelling (ASL) with MRI to quantify regional cerebral perfusion in young healthy individuals (18–35 years) and observed negative associations between AD-polygenic risk and regional perfusion, as well as lower CBF in those who possess a copy of the *APOE-ε*4 allele. Our findings suggest that vascular alterations in those with a broad increased genetic risk for AD manifest decades prior to symptom onset (Chandler et al., 2019). While our prior work provided insight into the influence of genetic risk factors on the cerebrovasculature in early adulthood, it is not yet known whether the influence of AD genetic risk scores on grey matter cerebral blood flow (GM CBF) remains consistent across the lifespan.

In the current study, we aim to determine the impact of AD risk alleles on CBF in an older population (mean age = 70). We anticipate that the combined influence of AD risk alleles will be associated with a reduction in global CBF (similar to our findings in (Chandler et al., 2019). Here, one predicts that either (1) the effects of AD risk alleles on CBF remain consistent or (2) demonstrate a more pronounced influence later in life. As AD risk alleles are likely to confer susceptibility by influencing expression of proximal genes, we further anticipate that regional CBF is spatially related to the transcript expression of these AD risk alleles. Here, we aim to take advantage of the Allen Human Brain Atlas (AHBA), a gene expression atlas that has advanced the development of imaging transcriptomics; linking macroscale brain imaging data to molecular function (for overview, see (Arnatkevic Iute et al., 2019; Fornito et al., 2019)). Prior evidence has revealed regional variations in gene expression with both functional connectivity with resting-state MRI (Forest et al., 2017), and structural connectivity with tractography (Goel et al., 2014). Yet, the link between AD risk alleles, physiological MRI measures, including regional perfusion, and the regional co-expression of AD risk gene transcripts proximal to these AD risk loci remains to be examined.

In order to address this hypothesis, we probe the AHBA to understand the relationship between AD risk gene expression and regional CBF across the cortex to determine if the influence of AD risk alleles can be explained by the regional co-expression of gene transcripts proximal to these AD risk loci (Arnatkevic Iute et al., 2019). Specifically, we sought to investigate whether brain-wide AD-related gene expression correlates with regional variation in CBF. While prior investigations have determined associations between gene expression and MRI based markers of regional aging / AD – associated atrophy, this approach has not yet been considered for cerebrovascular architecture (Groot et al., 2021; Vidal-Pineiro et al., 2020; Zhang et al., 2021). These analyses will establish the regional cortical co-expression of AD risk genes and AD-risk gene related CBF reductions, providing a plausible mechanistic link between AD risk loci and a well-established pathophysiological process in AD.

## 2 Methods

### 2.1 Participants

#### 2.1.1 ADNI sample

A total of 79 participants, classified as either healthy controls or having mild cognitive impairment took part in a series of MRI scans as part of their involvement in the ADNI protocol. To avoid population stratification issues between our AD GWAS - AD-GRS, and to compare to our prior sample (Chandler et al., 2019), we matched our test sample with demographically similar characteristics to the discovery sample (Kunkle et al., 2019), we included participants who self-reported as ‘White’ and ‘Not Hispanic or Latino’. Participants were further removed if they also contributed to discovery IGAP AD GWAS (N_OVERLAP_ = 2). Some participants were scanned at several time points, where the final number of discrete data points N_OBSERVATIONS_ = 127, where N_PARTICIPANTS_ = 44 completed 1 scan and N_PARTICIPANTS_ = 22 completed 2 scans and N_PARTICIPANTS_ = 13 completed 3 scans in the final analysis. See Table 1 for further demographic information.

#### 2.1.2 Cardiff sample

our younger sample was identical to our previous sample (Chandler et al., 2019) and consisted of 75(N_FEMALE_ = 47), righthanded individuals of western European descent, aged between 18–35, with at least 15 years of education. For further sample characterization (including ethics, exclusion criteria, genotyping methods, see Chandler et al., (2019).

### 2.2 Creation of polygenic scores

Polygenic score calculations were performed according to the procedure described by the International Schizophrenia Consortium, using the –score command in PLINK, via a wrapper function provided in the PRSice v1.25 software package (Euesden et al., 2015). Training data were from a recent AD GWAS (Kunkle et al., 2019), where SNPs were removed from summary statistics / geno-type data if they had a low minor allele frequency (*p* < 0.01) and data were pruned for linkage disequilibrium, removing SNPs within 500 kb and *R2* > 0.1 with a more significantly associated SNP. For the creation of the AD-GRS, we considered SNPs that were associated with AD that surpassed the GWAS threshold (PT < 5 ×10–8), as performed in and to make comparable to our original study (Chandler et al., 2019). We also removed all SNPs from the *APOE* gene on chromosome 19, as previously recommended (Ware et al., 2020) and individual *APOE ε*4 status was independently modelled in all analyses. Twenty-three SNPs were considered in the final AD-GRS calculation (see Fig. 2). To minimize potential confounding from population stratification linked to AD-GRS, we included the first 5 principle components from a linkage-disequilibrium (LD) pruned version of the genotypes as covariates in all analysis (Choi et al., 2020).

### 2.3 Imaging procedures and analysis of CBF

#### 2.3.1 ADNI sample

A 3T siemens PICORE MRI sequence (Wong et al., 1997) with pulsed ASL (or Q2TIPS) (Luh et al., 1999). The sequence parameters include repetition time (TR) = 3400 ms, echo time (TE) = 12 ms, TI1 = 700 ms, TI2 = 1900 ms, field of view (FOV) = 256 mm × 256 mm, number of slices: 24 axial, slice thickness = 4 mm, and image matrix size = 64 × 64. Pre-processing steps were conducted in SPM8 and included motion correction of individual ASL frames by rigid body transformation and least squares fitting. To obtain perfusion weighted images, the ASL data were then split into tag and control images and the mean-untagged data were sub-tracted from the mean-tagged data. The first volume of the ASL scan was used in place of an M0 (providing fully relaxed signal) to estimate blood-water-density proxy and used for calibration. A 3D MPRAGE T1-weighted sequence was collected for registration with the following parameters: TR = 2300ms, TE = 2.98ms, TI = 900ms, 176 sagittal slices, FOV = 256 × 240mm2, voxel size = 1.1 × 1.1 ×1.2mm3, flip angle = 9°. The perfusion data were registered to T1 space and rescaled to obtain CBF in ml/100g/min. For both samples, GM CBF values were sampled in native space across 82 cortical and subcortical parcellations as segmented using a FreeSurfer template (Desikan et al., 2006; Potvin et al., 2017). Full analysis including details of distortion correction, registration and partial volume correction can be found via the ADNI web page (http://adni.loni.usc.edu).

#### 2.3.2 Cardiff sample

Imaging data were collected on a 3T General Electric (GE) MRI scanner. Anatomical T1-weighted images were acquired with a 3D fast spoiled gradient echo sequence (FSPGR). Sequence parameters included: 172 contiguous sagittal slices with a slice thickness of 1 mm, TR = 7.9, TE = 3ms, inversion time of 450ms, flip angle = 20°, a FOV of 256 ×256 ×176 mm, matrix size 256 ×256 ×192 to yield 1 mm isotropic voxel resolution images. Resting CBF data were collected using a pseudo-continuous arterial spin labelling (PCASL) sequence. The study consisted of a single MRI session (which also comprised other functional and structural scans), and the PCASL sequence that lasted approximately 6 minutes. A PCASL sequence was acquired and included a 3D fast spin echo (FSE) spiral multi-slice readout. The sequence parameters included: number of excitations = 3, time to echo=32ms, echo time train length =64, TR =5.5seconds, matrix size =48 ×64 ×60, FOV =18 ×23 ×18cm, tag = 1500ms, PLD = 1500ms. The pcASL pre-processing for the sample can be found in (Chandler et al., 2019). Briefly, structural T1-weighted FSPGR images were registered to the M0 image acquired as part of the calibration of the CBF image acquisition, generating a transformation matrix. This transformation matrix was then applied to the skull stripped FSPGR (with reference/warping to the M0) using FSL’s Brain Extraction Tool. Next, linear registration via FSLs FLIRT registered the skull stripped anatomical image to the M0 transformation matrix (Montreal Neurological Institute (MNI) space) and the difference was calculated between this and the subject’s native space, providing data in the same space as the CBF data. The two transformation matrices for each participant were then concatenated to produce a matrix for the low resolution CBF image. All CBF images were then warped to standard MNI template using FSLs FLIRT. The priors for the grey matter were then registered to the skull stripped M0 image, generating a mask of grey matter from which CBF values was extracted.

### 2.4 Gene expression analysis

Publicly available human gene expression data from six post-mortem donors (N_FEMALE_ = 1), aged 24–57 (42.5 ± 13.38) were obtained from the Allen Institute (Hawrylycz et al., 2012). Data reflect the microarray normalization pipeline implemented in March 2013 (http://human.brain-map.org) and analyses were conducted according to the guidelines of the Yale University Human Subjects Committee. Normalized brain-wide gene transcript expression was mapped to 82 cortical /subcortical regions of interest as defined by the Desikan-Killiany atlas in abagen v0.0.3 (Arnatkevic Iute et al., 2019). Comprehensive processing details for the gene expression pre-processing and analysis can be found at https://abagen.readthedocs.io/en/stable/user_guide/reporting.html. In order to quantify AD risk gene transcript expression, we performed principal component analysis (PCA) for regional transcript expression. We identified two principal modes of covariation (see Fig. 4A–B) which were then individually regressed against regional CBF for the Cardiff and ADNI samples.

### 2.5 Statistical analysis

To maximize consistency of regression models across the samples, we included the same covariates for both sample analyses. Predictors were regressed against (1) whole GM CBF and (2) regional GM CBF for the 82 cortical / subcortical regions as defined by the Desikan-Killiany-Tourville (DKT) Atlas (Potvin et al., 2017). Regional CBF was z-normalized where each ROI was de-meaned and divided by the sample standard deviation to make comparison across ROIs and sample easier to interpret. The fixed effects of AD-GRS (P_T_ < 5 ×10^-8^) and *APOE* (number of *ε*4 alleles (0/1/2)) were modelled while controlling for age, biological sex, education, ICV, and the first 5 genetic principal components, acquired via the LD-pruned datasets. For the ADNI sample, we further included fixed effect covariates for (1) diagnostic status (modelling both a) healthy control / mild cognitive impairment and b) progression from healthy control to CI); (2) years of education; (3) cognition (as measured via the Montreal Cognitive Assessment Score); (4) site; (5) visit code (1) 12 months; 2) 24 months), and nested random effects for both (3) visit code and (4) subject, modelled as repeated measures. We included multiple time points to maximize sample size / statistical power. We employed outlier labelling / detection (Hoaglin and Iglewicz, 1987), which defines outliers using the interquartile range outlier labelling rule (1.5 × interquartile range (Q3-Q1)). This approach dynamically removed data points for each GM CBF dependent variable to minimize the impact of outlier data points. We compared the (1) CBF and (2) beta coefficients for the AD-GRS fixed effect for each region of interest between the Cardiff and ADNI samples using simple pearson r correlation. To control for false positives in each analysis, we compare each correlation to (1) 10,000 generated regional gene expression profiles equaling the number of genes used to generate the average AD gene expression (N_UNIQUE-GENES_ = 16) and (2) generated surrogate brain maps to simulate 10,000 null effects for CBF across 82 brain regions. To preserve potential autocorrelation across the brain maps and test for spatial specificity, we performed null spins of the brain parcellations to generate 10,000 surrogates for each map to create our null distributions (Alexander-Bloch et al., 2018; Burt et al., 2020; Wei et al., 2021). To assume that the strength of the gene expression – CBF covariation is more pronounced than expected by chance, the observed z-transformed correlation must surpass the alpha tail (Z > 1.96 / 95% CI: two-tailed) for the simulated distributions.

## 3 Results

### 3.1 Cerebral blood flow across the lifespan

**Fig. 1:**
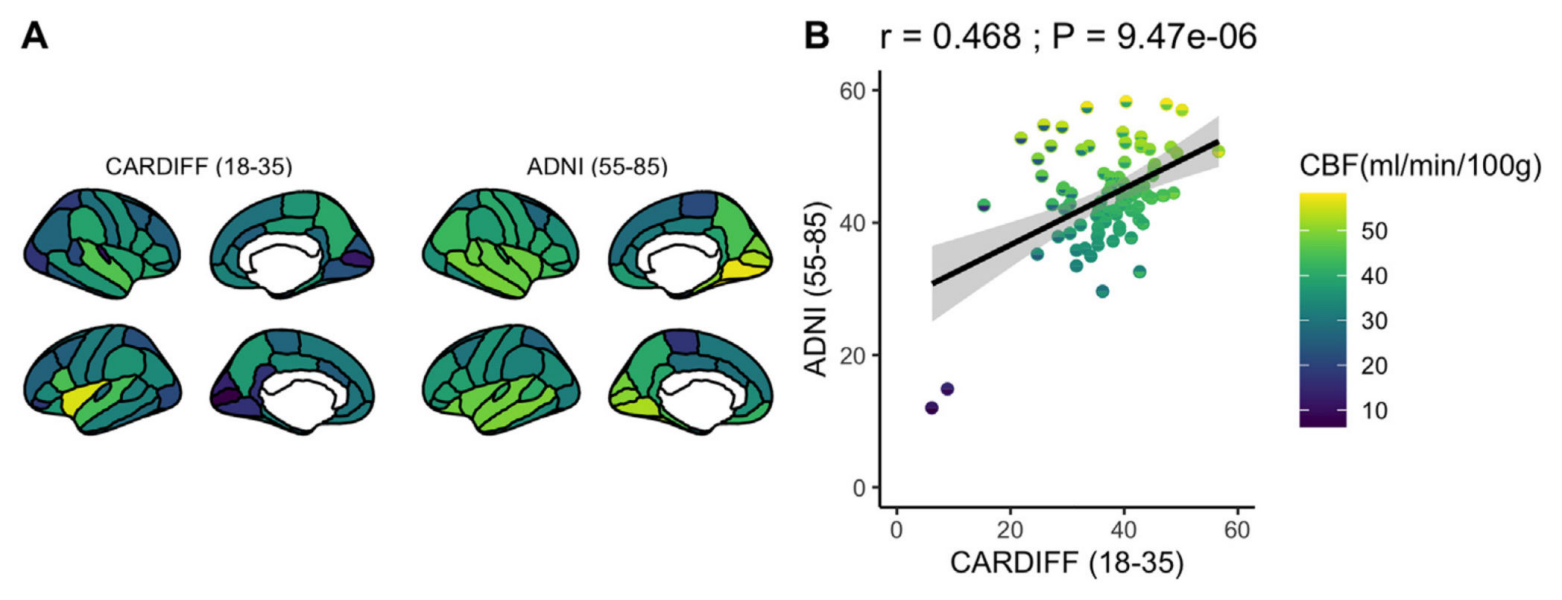
Regional GM CBF (ml/100g/min) in (A) the younger sample (aged: 18-35) previously described in Chandler et al., 2019 and (B) an older sample (aged: 55-85) and (C) Regional GM CBF (ml/100g/min) comparison for young (x-axis) and old (y-axis) across 82 cortical / subcortical regions.

First, we observed that regional GM CBF showed a consistent pattern of positive covariation between the younger and older sample (Fig. 1A-B), where cortical regions that showed higher per-fusion (ml/100g/min) in the younger sample was also comparably higher in the older sample (r = 0.468: *p* < 0.001; Fig. 1C), suggesting a pattern of consistent, regional variation in GM CBF across the lifespan.

### 3.2 AD-GRS effects on whole brain cerebral blood flow (ml/min/100g)

Similar to our original discovery (Chandler et al., 2019), we observed a significant negative association between whole brain GM-CBF and AD-GRS in the older (55 –85 years) ADNI sample (*β* = - 0.26; *p* = 0.011) after controlling for all covariates. Unlike our observation in younger individuals (Chandler et al., 2019) we did not observe a significant association between *APOE ε*4 absence / presence and whole brain GM CBF in the older sample (*β* = 0.28; *p* = 0.107). For all fixed effects and confidence intervals observed in the whole brain GM CBF analysis, see Table 2.

**Fig. 2:**
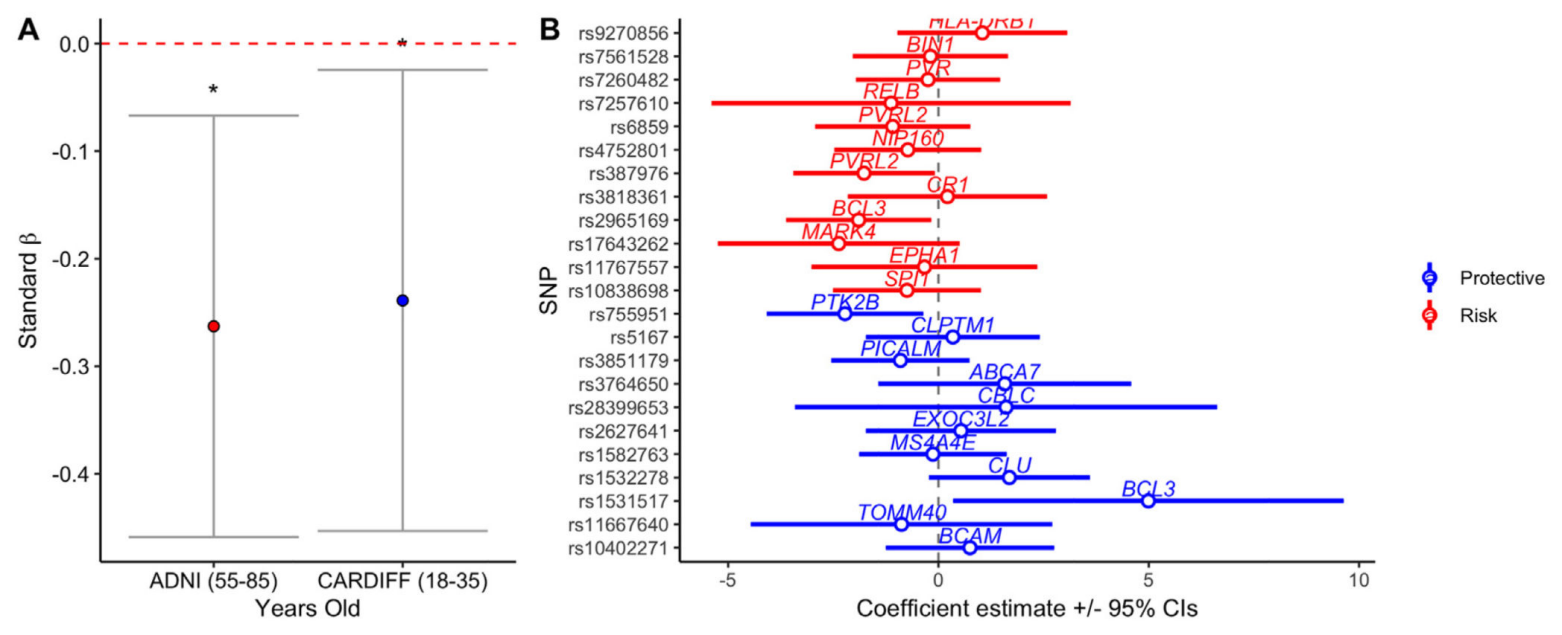
(A) Standardized AD-GRS effects on whole brain GM CBF for the young (18-35) and older (55-85) samples, * indicates *p* < 0.05, error bars represent 95% confidence intervals. (B) Diagnostic plot, demonstrating individual effects of AD risk (red) and protective (blue) SNPs on whole brain GMCBF, controlling for covariates in the older sample (55-85 years old). Circles / lines represent adjusted effect sizes and 95% confidence intervals. (For interpretation of the references to color in this figure legend, the reader is referred to the Web version of this article.)

To assess the individual impact of each of the SNPs in our AD-GRS model, we performed a linear regression analysis where each individual SNP was regressed in an additive model against whole brain GM CBF, controlling for all aforementioned covariates. Consistent with broad polygenic modelling assumptions, we observed a general propensity for SNPs that increase risk for AD (odds ratio (OR) > 1) to associate with reduced whole GM CBF, while alleles that conferred relative protection (OR < 1) for AD where associated with an increase in whole GM CBF (Fig. 2; sign test for direction of effects: *p* = 0.041).

### 3.3 Comparing AD-GRS effects on cerebral blood flow in early adulthood and older age

**Fig. 3:**
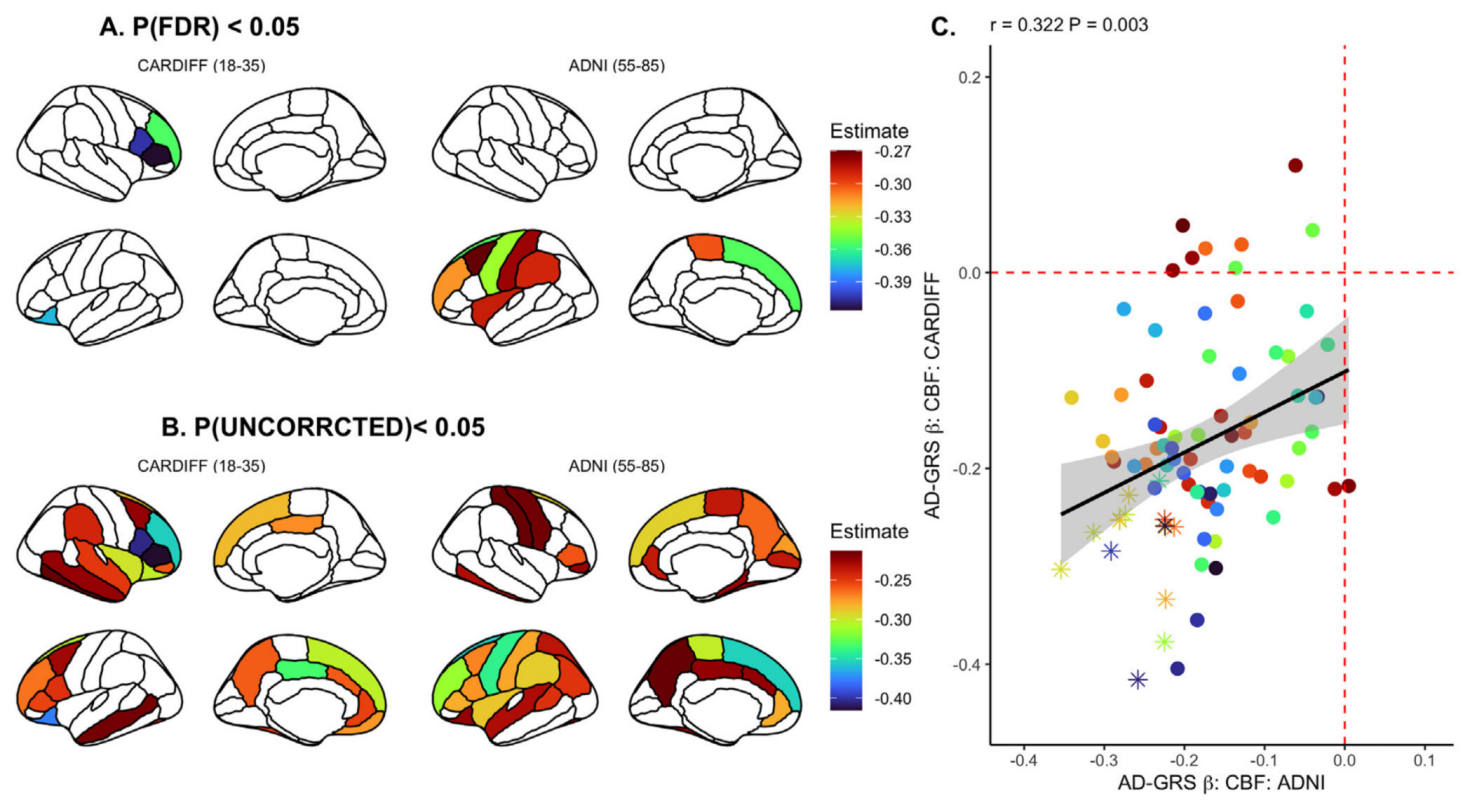
Regional association between AD-GRS and CBF in both Cardiff (Chandler et al., 2019) and ADNI samples, corrected for false discovery rate (A) (P_FDR_ < 0.05) and uncorrected (B) (P_UNCORRCECTED_ < 0.05). (C) Linear relationship of effect sizes (standardized beta coefficients) across the brain when comparing all cortical regions between sample B & C, where data points represented as an asterisk reflect *p* < 0.05 in both samples. Each point in the scatter plot represents one cortical / subcortical region. Regression slope grey area represents 95% confidence intervals.

As we observed an association between AD-GRS and whole brain GM CBF for both younger and older samples (Fig. 3a), we proceeded to explore the association at a regional level. We repeated the linear mixed-model analysis across 82 cortical / subcortical regions. Building upon our initial analysis in the younger sample (Chandler et al., 2019 replotted here in Fig. 3b), we observed a relationship between regional effect sizes across the brain, where the most / least pronounced effects of AD-GRS were comparable between young and older samples (Fig. 3d). In the ADNI sample of older individuals (55-85 years old) we found several specific regions with significant effects after correcting for false discovery rate, specifically within left frontal cortices (see Fig. 3, Supplementary Table 2). We did not observe the influence of *APOE ε*4 status on (1) whole brain GM CBF in the older sample and (2) a regional effect of *APOE ε4* on the younger sample, so did not proceed to investigate similarity between samples for *APOE ε4* GM CBF effects at a regional level (see Supplementary Table 3). For additional comparability with the Cardiff sample, we repeated the ADNI sample analyses, restricting to a single time point per participant, reflecting their latest scan (N = 79). We observed comparable associations between whole brain CBF and AD-GRS (*β* = - 0.265; *p* = 0.034) and a similar profile of regional spatial similarity with the Cardiff sample (r = 0.22; *p* = 0.042). However, we did not observe any individual, regional associations after FDR correction (lowest P_FDR_ = 0.054; left superior frontal gyrus).

### 3.4 Regional AD risk gene expression overlap

**Fig. 4:**
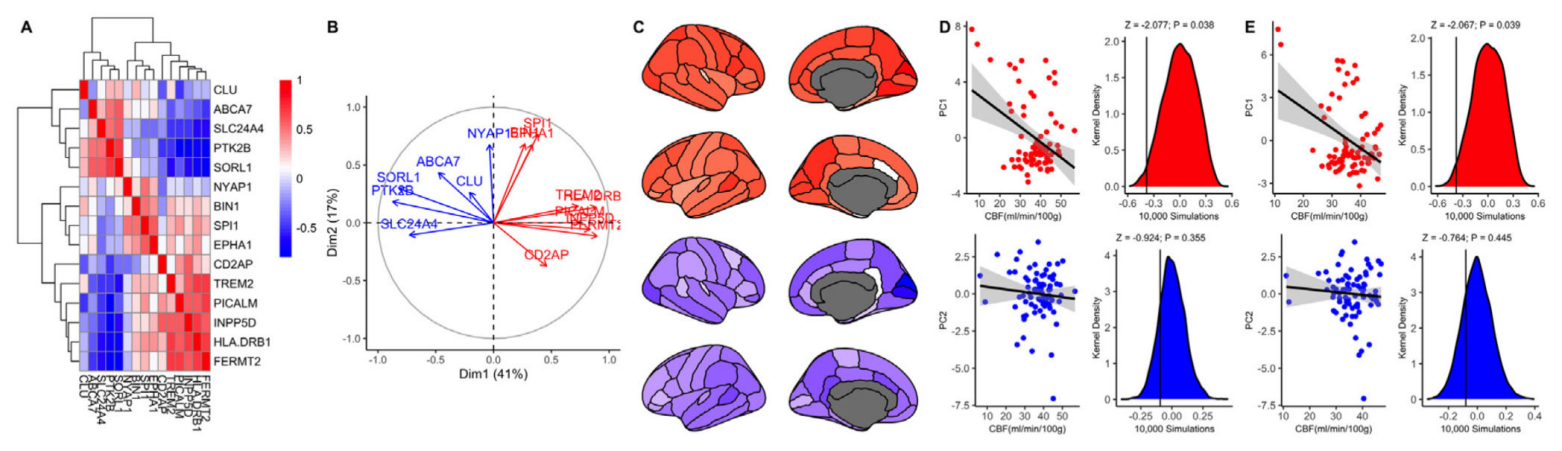
(A) Correlation matrix showing expression of AD risk genes. (B) Principal component analysis identified two principal modes of covariation between expression of all AD risk genes across the brain. (C) PC1-2 mapped onto the cortical regions. (D-E). Scatter plots show relationship between regional AD gene expression for PC1 (upper) and PC2 (lower) and regional CBF for the (D) Cardiff sample and (E) the ADNI sample. (D-E) Density plots for the distribution of 10,000 randomly simulated regional values (scaled to CBF range) for PC1-2 for Cardiff (D) and ADNI (E) samples. Solid black vertical lines represent the actual, observed correlation between PC1/2 AD gene expression and regional CBF.

We calculated the principal modes of covariation for transcript expression of AD risk genes proximal to the 23 SNPs used in AD-GRS model (N_UNIQUE-GENES_ =16) for the 82 cortical / subcortical regions (Fig. 4A). We correlated AD risk gene expression with (1) regional CBF (ml/100g/min) for the younger/older samples. We observed that mean AD gene expression was negatively associated with regional GM CBF in the young (Z = -2.07, *p* = 0.038) and older sample (Z = -2.067, *p* = 0.039) with no difference in correlation strength (z = -0.02, *p* = 0.98), suggesting that AD risk gene expression is highest in cortical regions where GM CBF is generally lower. We observed a similar pattern of association for *APOE* transcript expression and CBF, but these were not significant following the permutation testing (Cardiff: Z = -1.69, *p* = 0.091; ADNI: Z = - 1.30, *p* = 0.194, see supplementary Fig. 1).

## 4 Discussion

We sought to further investigate the impact of common AD genetic risk alleles on cerebral perfusion. Critically, the negative association we observed between AD-GRS and whole brain GM CBF in our prior work (Chandler et al., 2019) was also evident in the older population. This observation was further supported by evidence that regional effects of AD-GRS on GM CBF were correlated across samples. This suggests that the regional impact of AD-GRS on CBF remains consistent across the lifespan, and preferentially influences specific cortical structures previously implicated in preclinical models of AD related pathophysiology. Together these observations show that the cumulative impact of AD risk loci on this hallmark feature of AD pathogenesis is consistent across the lifespan.

The mechanisms by which common (e.g. intronic and intergenic) SNPs identified via GWAS confer susceptibility are largely unknown. However, a growing body of work suggests that these SNPs act as expression quantitative trait locus (eQTLs) and influence the expression of AD risk genes. Here, we tested the hypothesis that the brain-wide CBF variability would be spatially convergent with the expression AD risk gene transcripts. We found consistent regional covariation between mean AD risk gene expression and regional CBF perfusion pattern in both young and old samples. These findings demonstrate that the impact of AD-GRS on perfusion may confer susceptibility via the altered expression of proximal AD genes.

Cerebral blood flow shows a gradual and steady decrease across the lifespan (Bertsch et al., 2009; Devous et al., 1986; Hagstadius and Risberg, 1989; Heo et al., 2010; Lu et al., 2011). Here, our correlation demonstrates some evidence for spatial convergence of CBF variation between the young and old samples, suggesting that regional variability in GM CBF across the cortex remains somewhat consistent across age. It is not entirely understood why there is variability in GM CBF at rest across the brain. However, prior evidence has shown that brain perfusion closely correlates with brain function and metabolism (Detre et al., 2009), suggesting that variability in regional perfusion may reflect differences in energy demand across the cortex at rest.

In the second analysis, we showed that the association between regional CBF and AD-GRS in the young sample correlated positively with the association between regional CBF and AD-GRS in the older sample. The most significant effects were mostly observed in the frontal and temporal cortical structures. Critically, this result demonstrates that the AD-GRS effects seen in the older sample are regionally congruent with those in the younger sample. Our findings suggest that SNPs included in the AD genetic/polygenic risk model have consistent negative effects on cerebral perfusion from young adulthood and throughout the lifespan. In addition to AD-GRS we also investigated the effects of *APOE* on CBF across the samples. We saw no influence of *APOE ε*4 status on whole brain or regional CBF in the ADNI sample (unlike in our prior study (Chandler et al., 2019). These findings are consistent with the large studies which have observed limited association between CBF and *APOE* in middle / later life (Moonen et al., 2022). Research into the association between CBF and APOE across the lifespan remains mixed, with some studies demonstrating CBF reductions in vulnerable medial temporal lobe structures in AD (Rubinski et al., 2021). We suggest that while the AD-GRS influence on CBF across the lifespan remains consistent, *APOE* status may have a more dynamic role in shaping CBF which may change across the lifespan (Wierenga et al., 2013) and requires further investigation.

In our third analysis we used gene expression data to identify how AD risk genes expression across the cortex correlates with regional CBF. Our results show a negative association between ADgene expression and regional CBF, suggesting that AD risk genes may spatially covary with regional cerebral perfusion. Moreover, our findings demonstrate that the regional covariation between cerebral perfusion and AD gene expression occurs throughout the lifespan.

While amyloid and tau-genic hypotheses provide important insight into preclinical AD models (Bloom, 2014; Gotz et al., 2001; Ittner and Gotz, 2011; Lewis et al., 2001), vascular dysregulation occurs prior to this AD pathophysiology (Iturria-Medina et al., 2016). We provide additional support for vascular dysregulation and hypoperfusion as early markers of AD risk that may be observed during young adulthood. Moreover, we suggest that cerebral perfusion is a potentially important AD related pathological feature and should be considered as a target for therapeutic intervention.

Our observations should be considered with the following limitations. First, while we observed consistent AD-GRS effects across the lifespan, we did not observe *APOE* related effects in our older sample, so we cannot infer that all AD genetic effects were consistent. This may be explained by dynamic *APOE* effects that have recently been discovered in recent MRI studies, where *APOE* effects manifest in early life, for example in the volume of medial temporal lobe structures such as the amygdala (Brouwer et al., 2020). We therefore suggest our study warrants further exploration of APOE / CBF in larger samples across the lifespan. Second, there are several differences within and between these samples, including non-equivalent scanner sequence (pCASL / pASL), site / scanner differences and pre-processing pathways which makes direct comparison or a combined analysis challenging, and observations about sample consistency should be interpreted with caution. Third, while we observed an association between whole brain CBF and AD-GRS in the ADNI sample and similar pattern of alterations to the Cardiff sample, the specific regions were not the same and the impact of AD-GRS on specific brain regions should be considered tentative until replicated in an independent, demographically similar sample. The negative association between AD-GRS and regional CBF were also weaker in the cross-sectional analysis of the ADNI, further suggesting larger samples will be beneficial in confirming the spatiotemporal relationship between genetic risk for AD and CBF. Last, we acknowledge potential confounding from other AD-related pathophysiology / cere-brovascular risk factors (Amen et al., 2020; Broce et al., 2019; Korte et al., 2020; Rubinski et al., 2021). We suggest that future research should incorporate further longitudinal molecular imaging such as glucose metabolism, amyloid, and tau imaging as well as known cerebrovascular risk factors. These would help to determine the trajectory of whole brain and regional perfusion changes across the lifespan and its association to further oxygenation changes and metabolic dysfunction in those at risk of AD.

To conclude, we demonstrate a consistent negative influence of additive genetic AD risk on cerebral perfusion across the lifespan, which was also related to regional expression of proximal AD risk genes across the cortex. Thus, reduced CBF may be a central, and proximal, process in the pathophysiology of AD, and a potential mechanism by which AD risk genes exert their adverse effects on brain structure and function.

## Supplementary Material

Supp Fig 1

Supp Table 1

Supp Table 2

Supp Table 3

## Figures and Tables

**Table 1:** Sample characteristics.

	Participants with baseline scan	Participants with 12-mo follow-up scan	Participants with 24-mofollow-up scan	Overall
	(N = 44)	(N = 22)	(N = 13)	(N _TOTAL_=127, N_UNIQUE_ = 79)
Age				
Mean (SD)	71.0 (6.95)	68.3 (5.26)	67.2 (5.64)	68.9 (6.11)
Median [Min, Max]	70.3 [55.0, 83.4]	68.3 [58.5, 78.9]	65.5 [59.5, 78.5]	68.2 [55.0, 83.4]
Sex				
Female	19 (43.2%)	10 (45.5%)	6 (46.2%)	57 (44.9%)
Male	25 (56.8%)	12 (54.5%)	7 (53.8%)	70 (55.1%)
Diagnosis				
CN	13 (29.5%)	5 (22.7%)	5 (38.5%)	38 (29.9%)
EMCI	19 (43.2%)	13 (59.1%)	6 (46.2%)	63 (49.6%)
LMCI	12 (27.3%)	4 (18.2%)	2 (15.4%)	26 (20.5%)
APOE4				
0	27 (61.4%)	13 (59.1%)	12 (92.3%)	89 (70.1%)
1	17 (38.6%)	6 (27.3%)	0 (0%)	29 (22.8%)
2	0 (0%)	3 (13.6%)	1 (7.7%)	9 (7.1%)
Visit (Mos)				
6	21 (47.7%)	9 (40.9%)	8 (61.5%)	52 (40.9%)
12	16 (36.4%)	7 (31.8%)	2 (15.4%)	44 (34.6%)
24	7 (15.9%)	6 (27.3%)	3 (23.1%)	31 (24.4%)
HC > MCI (Conversion)				
0	43 (97.7%)	18 (81.8%)	12 (92.3%)	115 (90.6%)
1	1 (2.3%)	4 (18.2%)	1 (7.7%)	12 (9.4%)
MoCA				
Mean (SD)	24.6 (2.98)	25.3 (2.85)	25.6 (1.98)	25.2 (2.65)
Median [Min, Max]	25.0 [16.0, 30.0]	26.0 [18.0, 29.0]	26.0 [22.0, 30.0]	26.0 [16.0, 30.0]
Education (Yrs)				
Mean (SD)	16.5 (2.64)	17.4 (1.97)	17.5 (2.88)	17.1 (2.50)
Median [Min, Max]	16.0 [12.0, 20.0]	17.5 [14.0, 20.0]	18.0 [12.0, 20.0]	18.0 [12.0, 20.0]
Intracranial Volume (mm3)				
Mean (SD)	1,510,000 (140,000)	1,550,000 (163,000)	1,570,000 (182,000)	1,540,000 (160,000)
Median [Min, Max]	1,500,000 [1,290,000, 1,830,000]	1,550,000 [1,260,000, 2,070,000]	1,550,000 [1,260,000, 1,830,000]	1,530,000 [1,260,000, 2,070,000]

**Table 2:** Fixed effect predictors (*β* estimate and 95% confidence intervals) regressed against whole brain GM CBF in the final sample of ADNI participants controlling for the top 5 principal components (PCs) as additional covariates of no interest and visit code / subject as nested random effects.

FIXED EFFECTS	BETA	CI (2.5%)	CI (97.5%)	T-STATISTIC	DF	*p*-VALUE
(Intercept)	-1.19726	-3.21405	0.819521	-1.16353	76.11257	0.248248
AD-GRS	-0.26292	-0.45875	-0.06709	-2.63145	67.49825	0.010526
APOE4	0.289192	-0.05816	0.636544	1.631796	65.6209	0.107513
MCI	0.007101	-0.31093	0.325133	0.043761	110.8895	0.965174
MCI-CONVERSION	0.357973	-0.35038	1.066325	0.99049	61.00472	0.325847
AGE	0.018739	-0.00964	0.047115	1.294349	72.57036	0.199648
SEX(M)	-0.34147	-0.78345	0.100503	-1.51428	67.246	0.134642
SITE	0.000119	-0.0009	0.001139	0.229399	11.33588	0.822645
FOLLOW-UP (12M)	-0.49858	-0.69474	-0.30243	-4.98176	62.90319	5.21E-06
FOLLOW-UP (24M)	-0.82986	-1.04813	-0.6116	-7.45195	56.49852	5.9E-10
EDUCATION	0.031833	-0.16608	0.229751	0.315243	74.35791	0.75346
MOCA	0.062863	-0.09299	0.218718	0.790535	99.64165	0.431093
ICV	0.260453	0.04815	0.472756	2.404479	66.19241	0.019001

Key: AD-GRS, alzheimer’s disease genetic risk score; MCI, mild cognitive impairment; MOCA, montreal Cognitive Assessment; ICV, intracranial volume
