# Supplementary material for: Alzheimer’s genetic risk effects on cerebral blood flow across the lifespan are proximal to gene expression
Source: Neurobiol Aging. Author manuscript; Available in PMC 2023 Oct 2. (PMC7615143; doi:10.1016/j.neurobiolaging.2022.08.001)
Supplement: Supp Fig 1 [file EMS188115-supplement-Supp_Fig_1.docx]

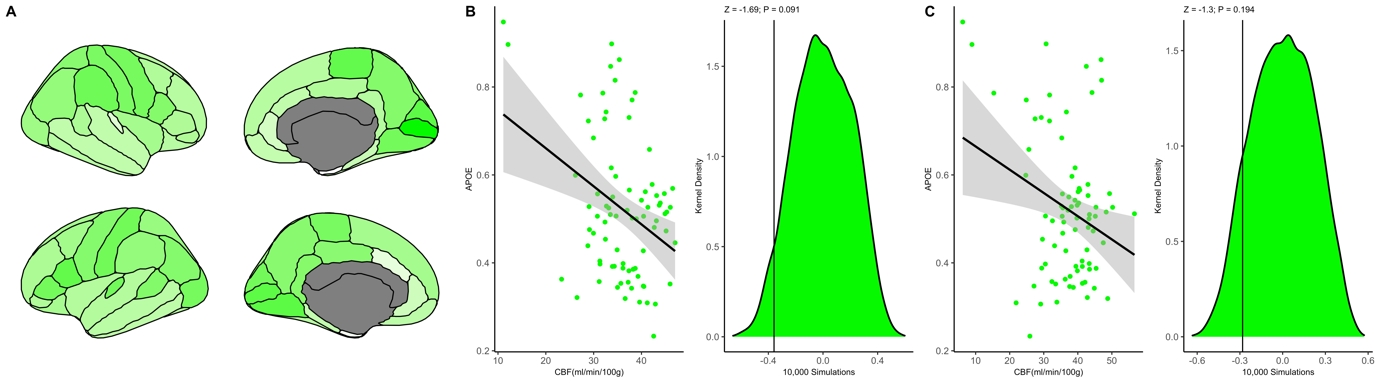


Supplementary Figure 1. A) *APOE* transcript expression mapped onto cortical regions. B-C) Scatter plots show relationship between regional *APOE* gene expression for regional CBF for the B) Cardiff sample and C) the ADNI sample. B-C) Density plots for the distribution of 10,000 simulated randomly simulated regional values (scaled to CBF range) for *APOE* for Cardiff (B) and ADNI (C) samples. Solid black vertical lines represent the actual, observed correlation between *APOE* gene expression and regional CBF.
